# Ratios in regression analyses with causal questions

**DOI:** 10.1093/aje/kwae162

**Published:** 2024-06-26

**Authors:** Sanne S Mooldijk, Jeremy A Labrecque, M Arfan Ikram, M Kamran Ikram

**Affiliations:** Department of Epidemiology, Erasmus MC - University Medical Center Rotterdam, 3000 CA Rotterdam, The Netherlands; Department of Epidemiology, Erasmus MC - University Medical Center Rotterdam, 3000 CA Rotterdam, The Netherlands; Department of Epidemiology, Erasmus MC - University Medical Center Rotterdam, 3000 CA Rotterdam, The Netherlands; Department of Epidemiology, Erasmus MC - University Medical Center Rotterdam, 3000 CA Rotterdam, The Netherlands; Department of Neurology, Erasmus MC - University Medical Center Rotterdam, 3000 CA Rotterdam, The Netherlands

**Keywords:** body mass index, causal inference, ratio, regression analyses, statistical interaction

Ratio measures are often used in medical research. Generally, the reason is to describe a phenomenon with a single number rather than multiple numbers. In many instances, ratios are used to account at an individual level for size, such as height in body mass index (BMI) or volume in concentrations. Alternatively, they are used to quantify a latent construct or to summarize the balance between two phenomena, for example, of two hormones or other laboratory markers,^1^ as the two measures jointly are thought to better describe a phenomenon than separately.

Despite several statistical issues previously described, such as finding spurious correlations,^2^^-^^4^ ratio measures can be convenient and efficient when it comes to diagnostic, prognostic, or predictive questions. However, when dealing with causal questions, the use of ratio measures is not straightforward. An example where a ratio has led to misinterpretation relates to the retinal arteriolar-to-venular ratio, initially thought of as a measure for arteriolar narrowing. Several associations with cardiovascular phenomena were found, but these later appeared to be partly attributable to larger venular diameters.^5^

We explore several issues relevant for the use of ratios within a causal framework. These issues stem partly from the fact that once two variables are combined into a ratio, the individual effects of the components cannot be distinguished. This is because ratios are essentially terms for interaction between one variable and the reciprocal of another variable: *X*/*Z* = *X* × *Z*^-1^. We discuss three situations in which a ratio measure can be used: as an exposure, a confounder and an outcome.

## Ratio as exposure

A typical model to estimate the effect of a ratio, for example BMI, on an outcome *Y* could be:


(1) \begin{equation*} Y={\mathrm{\beta}}_0+{\mathrm{\beta}}_1\frac{Weight}{Height^2}+{\mathrm{\beta}}_2 covariate+\mathrm{\varepsilon} \end{equation*}

Here, BMI here is a term for the interaction between weight (in kilograms) and height (in meters) (corresponding causal model in Appendix S1).

A first problem that is created in this situation relates to the consistency assumption, requiring that an exposure be well-defined, leaving no ambiguity about the counterfactual value of the outcome if we intervene on the exposure. In the case of a ratio as exposure, how do we know whether the causal effect of changing a ratio is due to changing the numerator, the denominator, or both? For example, if we wanted to know a person’s risk of mortality if we increased their BMI, this would not meet the consistency assumption because the risk would almost certainly be different had we increased their weight rather than gruesomely decreased their height. The purpose of dividing by height is generally to correct for body size. Therefore, it is better to think of weight as the exposure (acknowledging that weight itself does not, per se, meet the consistency assumption).^6^

A second problem is that a strong parametric assumption is made about the relationship of the components of the ratio with the outcome. In equation 1, the interaction between weight and height restricts the effect of weight to be inversely related to height, and because there is no main effect for weight or height, it assumes that weight and height have no effect on the outcome except through the interaction term. To model BMI in this way, one has to be very sure that this is the correct relationship between weight, height, and the outcome, namely that the effect on the outcome of increasing weight by 1 kilogram can only become smaller with increasing height. Additionally including terms for weight and height (equation 2) allows for more flexibility in modeling the outcome *Y* (as shown in Figure S1).


(2) \begin{equation*} {Y={\mathrm{\beta}}_0+{\mathrm{\beta}}_1 Weight+{\mathrm{\beta}}_2{Height}^{-2}+{\mathrm{\beta}}_3\frac{Weight}{Height^2}+{\mathrm{\beta}}_4 covariate+\mathrm{\varepsilon} } \end{equation*}

In many instances, weight will drive associations of BMI with an outcome, and height might act as a confounder or effect modifier.^7^ Here, we illustrate an example of BMI as exposure and systolic blood pressure (SBP) as outcome, adjusted for age and use of antihypertensive medication:


(3) \begin{equation*} { SBP={\mathrm{\beta}}_0+{\mathrm{\beta}}_1\frac{Weight}{Height^2}+{\mathrm{\beta}}_2 Age+{\mathrm{\beta}}_3 Antihypertensives+\mathrm{\varepsilon} } \end{equation*}

Commonly, the ratio components are first included as separate terms in the model and afterwards their interaction is investigated:


(4) \begin{align*} SBP&={\mathrm{\beta}}_0+{\mathrm{\beta}}_1 Weight+{\mathrm{\beta}}_2{Height}^{-2}+{\mathrm{\beta}}_3\frac{Weight}{Height^2}\notag\\&\quad+{\mathrm{\beta}}_4 Age+{\mathrm{\beta}}_5 Antihypertensives+\mathrm{\varepsilon} \end{align*}

We fitted various regressions with cross-sectional data from 5922 participants of the Rotterdam Study.^8^ The coefficients resulting from linear regressions with terms for weight, height, and/or BMI, all adjusted for age and use of antihypertensive medication, are shown in Table 1. The results suggest effect modification: The effect sizes differ for varying heights. It appears that different model specifications may lead to varying conclusions about the effect of weight at different heights. For instance, model 1, with solely a term for BMI, suggests that a 1-unit increase in body weight has a larger effect on SBP for a small person than for a tall person. Models 4, 5, and 6 (including separate terms for weight, height, and their interaction) show the opposite. Importantly, in models with BMI, the coefficients for weight and height as main effects are not equal to zero. Thus, these terms should not be omitted, which is typically the case when only BMI is included in a model.

**Table 1:** Results from the association of adiposity with systolic blood pressure in the population based Rotterdam Study.

**Model^**a**^**	**Estimated coefficients (95% CI)**					**Effects of weight conditional on height** ^**b**^ **(95%CI)**		
	**Weight**	**Height**	**Height** ^**-2**^	**Weight × height**	**Weight × height** ^**-2**^	**Height of 1.57 m**	**Height of 1.68 m**	**Height of 1.82 m**
1. SBP ~ weight × height^-2^					0.54 (0.41 to 0.66)	0.22 (0.17 to 0.27)	0.19 (0.15 to 0.23)	0.16 (0.13 to 0.20)
2. SBP ~ weight	0.13 (0.09 to 0.16)					0.13 (0.09 to 0.16)	0.13 (0.09 to 0.16)	0.13 (0.09 to 0.16)
3. SBP ~ weight + height	0.20 (0.15 to 0.24)	−19.23 (−25.89 to −12.57)				0.20 (0.15 to 0.24)	0.20 (0.15 to 0.24)	0.20 (0.15 to 0.24)
4. SBP ~ weight + height + weight × height	−0.33 (−0.86 to 0.20)	−44.12 (−70.03 to −18.21)		0.31 (0.00 to 0.62)		0.16 (0.10 to 0.22)	0.19 (0.15 to 0.24)	0.23 (0.18 to 0.29)
5. SBP ~ weight + height + weight × height^-2^	0.67 (0.31 to 1.02)	−63.21 (−97.13 to −29.29)			−1.33 (−2.34 to −0.32)	0.13 (0.06 to 0.20)	0.20 (0.15 to 0.24)	0.26 (0.20 to 0.33)
6. SBP ~ weight + height^-2^ + weight × height^-2^	0.42 (0.15 to 0.69)		95.11 (34.27 to 155.96)		−0.64 (−1.41 to 0.12)	0.16 (0.10 to 0.22)	0.19 (0.15 to 0.24)	0.22 (0.17 to 0.28)

Abbreviation: SBP, systolic blood pressure.

^a^Height in meters, weight in kilograms, and SBP in mm Hg. All models adjusted for age and for use of antihypertensive medication. For this example, we used data from the Rotterdam Study^8^ subcohorts RS-I (5th visit), RS-II (3rd visit), RS-III (2nd visit).

^b^The effects of weight conditional on height are marginal effects (with all other covariates except for height set at their mean) for persons with a height equal to the 10th percentile (1.57 m), the median (1.68 m), or the 90th percentile (1.82 m). The numbers indicate a difference in SBP in mm Hg per 1-unit increase in weight in kilograms.

## Ratio as covariate

Ratios are also used to adjust for confounding, such as *Z*_2_/*Z*_3_ in the following regression:


(5) \begin{equation*} {\displaystyle \begin{array}{c}Y={\mathrm{\beta}}_0+{\mathrm{\beta}}_1{X}_1+{\mathrm{\beta}}_2\ {Z}_2/{Z}_3+\mathrm{\varepsilon} \end{array}} \end{equation*}

Here, our primary interest is the effect estimate of the exposure (β_1_) rather than effect estimates of the ratio and its components. Although perhaps uncommon, it is possible for the interaction between two variables to also be a confounder beyond the confounding effect of the ratio components. In such cases, the interaction must therefore be adjusted for, in order to remove the confounding stemming from it. When only the ratio between two variables is included as a covariate, a strong statement is being made that only the interaction between two variables confounds the causal effect being studied and not the main effects of the variables. For instance, adjusting for the ratio between total cholesterol and high-density lipoprotein cholesterol, but not for the variables separately, assumes that their confounding effects are fully captured by the ratio. It may also be possible that a ratio may better capture confounding effects than separate linear terms, for example, if certain transformations of the components are used that better describe a confounding effect, eg, height^2^ instead of height. Thus, including a ratio as an independent variable in a regression with the aim of adjusting for confounding does not necessarily lead to misinterpretation or bias.

## Ratio as outcome

Ratios may also be used as the outcome, or dependent variable, in regressions. A simple regression with *X* as the determinant and *Y*/*Z* as the outcome of interest may be:


(6) \begin{equation*} {\displaystyle \begin{array}{c}Y/Z={\mathrm{\beta}}_0+{\mathrm{\beta}}_1X+{\mathrm{\beta}}_2 covariate+\mathrm{\varepsilon} \end{array}} \end{equation*}

An effect of *X* on *Y*/*Z* can be found if *X* causes either *Y* or *Z*, or both. The interpretation of coefficient β_1_ is thus complicated since it always comprises effects on changes in both *Y* and *Z*. Further challenges of these models become clear if both sides of the equation are multiplied by *Z*:


(7) \begin{equation*} {\displaystyle \begin{array}{c}Y={\mathrm{\beta}}_0Z+{\mathrm{\beta}}_1 XZ+{\mathrm{\beta}}_2Z\ covariate+\mathrm{\varepsilon} Z\end{array}} \end{equation*}

Regression 7 lacks both an intercept for *Y* and a main effect of *X*. Instead, *X* only appears in an interaction term together with variable *Z*, and so we essentially study the effect of an interaction between *X* and *Z* on *Y*. In other words, it is assumed that *Y* is not related to *X*, except through the interaction of *X* with *Z*. The equation also shows that any unexplained variance in *Y* depends not only on epsilon, but also on the value of *Z*. Although it is theoretically possible that this model correctly describes the relations between *X*, *Y*, and *Z*, in many instances, this may not be the case, and consequently the estimated coefficients for effects of *X* will be biased. It is important to consider what the most suitable formulation of the regression model is to answer the question of interest. These choices should depend on the assumed causal links between variables in the model, based on existing knowledge. For example, in the case where *Z* might be an effect modifier of the effect of *X* on *Y*, a solution could be to include it as an interaction term with *X*.

BMI can again be an example of a ratio used in an attempt to correct for size, this time as the dependent variable (*Y*/*Z*). According to equation 6, β_1_ would refer to the increase in kg/m^2^ per unit increase in *X*, if the covariates were kept constant. This is generally interpreted as a change in adiposity, but could just as easily be the result of a change in height due to its association with *X*, which can particularly be relevant in children or in elderly.

When studying effects on a ratio that reflects a certain balance, say between total and high-density lipoprotein cholesterol, or between the innate and adaptive immune system, the magnitude of β_1_ is generally of little biological value. Moreover, relevant information regarding etiology with respect to the individual components of the ratio is lost. In situations where *X* is thought to have an effect on *Y* and *Z* separately, the causal questions may be better answered by analyzing those separately (ie, effect of *X* on *Y* and effect of *X* on *Z*) with additional adjustment for confounding.

## Conclusions

Ratios are widely used in regressions in medical research, including research with causal questions. Given that ratios are essentially interactions terms and used without their main effects, strong assumptions are made in the model formulation and further challenges emerge in the interpretation. For instance, important causal inference concepts, such as the consistency assumption, may be violated when including a ratio as the exposure. As in any study with a causal question, we recommend considering the causal structure of the studied variables, based on subject knowledge, to motivate the use or non-use of a ratio for model specification, instead of relying on ratios that may appear convenient in practical use but introduce potential issues with respect to causal inference.

## Supplementary Material

Web_Material_kwae162

## Data Availability

Data can be obtained upon request. Requests should be directed towards the management team of the Rotterdam Study (datamanagement.ergo@erasmusmc.nl), which has a protocol for approving data requests. Because of restrictions based on privacy regulations and informed consent of the participants, data cannot be made freely available in a public repository.
