# Supplementary material for: Ratios in regression analyses with causal questions
Source: Am J Epidemiol. 2024 Jun 26;194(1):311–3. doi: 10.1093/aje/kwae162 (PMC11735960; doi:10.1093/aje/kwae162)
Supplement: Web_Material_kwae162 [file web_material_kwae162.pdf]

Supplementary Material

Ratios in regression analyses with causal questions

Sanne S. Mooldijk, Jeremy A. Labrecque, M. Arfan Ikram, M. Kamran Ikram

Contents

Appendix S1 Causal model of the ratio as an exposure

Figure S1 The effect of weight on systolic blood pressure with varying weights according to different models

Appendix S1. Causal model of the ratio as an exposure

Imagine the following causal model:

$$E[Y^w|H,\boldsymbol{L}] = \beta_0 + \beta_w \cdot w + \beta_H \cdot H + \beta_{w,H} \cdot f(w,H) + \beta_{\boldsymbol{L}} \cdot \boldsymbol{L}$$

Where I is a counterfactual outcome when weight W is set to w, H is height and $\boldsymbol{L}$ is a vector of covariates. The causal effect of changing w^* to w is $\beta_w \cdot (w^* - w) + \beta_{w,H} \cdot \left(f(w^*,H) - f(w,H)\right)$. It would be a common choice to estimate the effect of W with body mass index (BMI) as the main exposure and adjusting for $\boldsymbol{L}$:

$$E[Y|W,H,\boldsymbol{L}] = \alpha_0 + \alpha_{BMI} \cdot \frac{W}{H^2} + \alpha_{\boldsymbol{L}} \cdot \boldsymbol{L}$$

However, the estimate from this model, α_{BMI}, would only equal the causal effect when $\beta_w = \beta_H = 0$ and $f(w,H) = \frac{w}{H^2}$. This demonstrates the strong parametric assumptions imposed when only including BMI, or any ratio, into a model. These parametric assumptions can easily be avoided, in the case of β_w and β_H by including main effects terms in the model. The assumption that $f(w,H) = \frac{w}{H^2}$ can be avoided by considering other forms of interaction for $f(w,H)$.

It should also be noted that the consistency assumption, $Y^w = Y$ for those with *W*=*w*, conditional exchangeability, $W \perp Y\^w|H,\boldsymbol{L}$, and positivity must be satisfied.

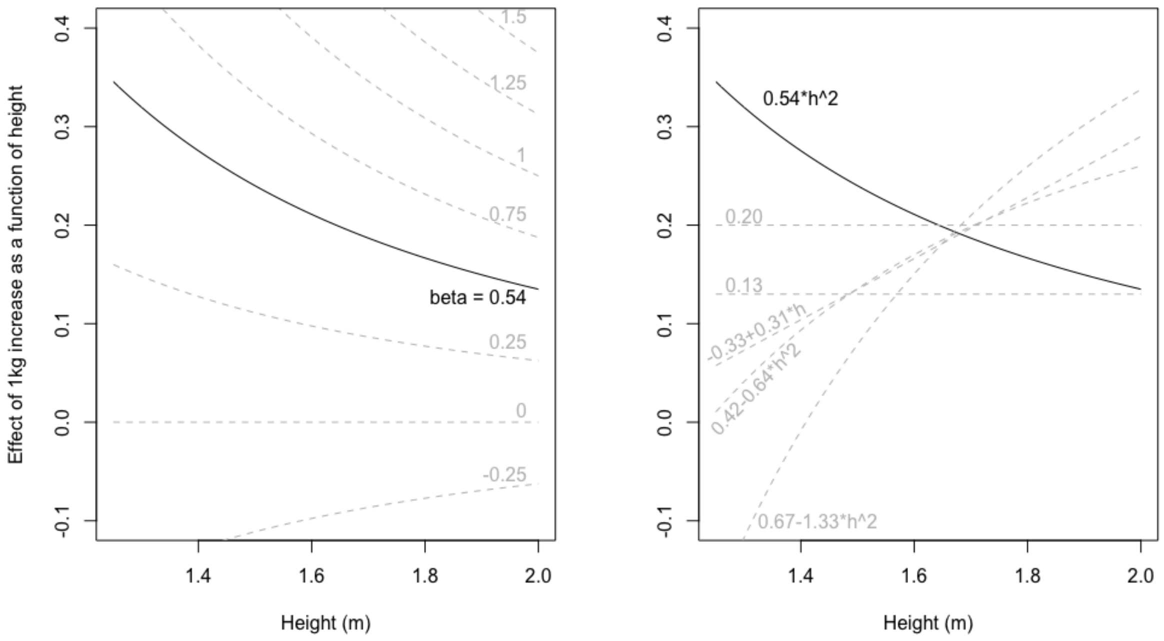

Figure S1. The effect of weight on systolic blood pressure with varying weights according to different models

The left panel plots the effect of increasing weight by one kilogram as a function of height. The black line is the estimated effect when only body mass index (BMI) is used in the model. The grey lines demonstrate the effect of weight if the coefficient for BMI was higher or lower. In the right panel, the black line again is the estimate effect of weight when only BMI is used in the model. The grey lines are the effect of weight for models 2 through 6 in Table 1. When additional terms for weight and an interaction between weight and height are included in the model, the effect of weight on the outcome as much more flexibility.
